# Selection and Characterization of Vimentin-Binding Aptamer Motifs for Ovarian Cancer

**DOI:** 10.3390/molecules26216525

**Published:** 2021-10-28

**Authors:** Andrea M. Costello, Miguel-Angel Elizondo-Riojas, Xin Li, David E. Volk, Anil K. Pillai, Hongyu Wang

**Affiliations:** 1 Institute of Molecular Medicine, McGovern Medical School, University of Texas Health Science Center at Houston, 1825 Hermann Pressler, Houston, TX 77030, USA; amcostello@houstonmethodist.org (A.M.C.); xin.li@uth.tmc.edu (X.L.); david.volk@uth.tmc.edu (D.E.V.); 2 Centro Universitario Contra el Cáncer (CUCC), Servicio de Oncología, Universidad Autónoma de Nuevo León, Hospital Universitario “Dr. José Eleuterio González”, Nuevo, León 64460, Mexico; riojas_miguel@hotmail.com; 3 Department of Radiology, University of Texas Southwestern Medical Center, Dallas, TX 75235, USA; anil.pillai@utsouthwestern.edu; 4 Department of Diagnostic and Interventional Imaging, McGovern Medical School, University of Texas Health Science Center at Houston, 6431 Fannin Street, Houston, TX 77030, USA

**Keywords:** aptamer, binding motifs, ovarian cancer

## Abstract

The application of aptamers in biomedicine is emerging as an essential technology in the field of cancer research. As small single-stranded DNA or RNA ligands with high specificity and low immunogenicity for their targets, aptamers provide many advantages in cancer therapeutics over protein-based molecules, such as antibodies. Vimentin is an intermediate filament protein that is overexpressed in endothelial cells of cancerous tissue. High expression levels of vimentin have been associated with increased capacity for migration and invasion of the tumor cells. We have selected and identified thioated aptamers with high specificity for vimentin using human ovarian cancer tissues. Tentative binding motifs were chosen for two vimentin aptamers based on predicted secondary structures. Each of these shorter, tentative binding motifs was synthesized, purified, and characterized via cell binding assays. Two vimentin binding motifs with high fidelity binding were selected and further characterized via cell and tissue binding assays, as well as flow cytometric analysis. The equilibrium binding constants of these small thioated aptamer constructs were also determined. Future applications for the vimentin binding aptamer motifs include conjugation of the aptamers to synthetic dyes for use in targeted imaging and therapy, and ultimately more detailed and precise monitoring of treatment response and tumor progression in ovarian pathology.

## 1. Introduction

The field of medicine is becoming increasingly more personalized with a focus on tailoring treatment to the specific needs of each patient to provide more efficient and goal-directed care. This is particularly relevant in the fields of oncology and rheumatology where targeted immunotherapy via the use of antibodies has emerged as an alternative to traditional chemotherapeutics. Aptamers, which are small, single stranded DNA or RNA oligonucleotides, serve as another avenue to achieve a more accurate therapeutic regimen and provide several advantages over the use of antibodies for targeted therapy. Aptamers are much smaller than antibodies [1], and therefore, are less immunogenic when introduced into a host [2,3]. Being that the aptamers are oligonucleotides, they are easily synthesized in a laboratory setting and are easily customizable for a variety of applications. The ease of synthesis and customization also allows aptamers to be widely used in a diagnostic setting, as they can be conjugated with dyes or radioisotope for use in radiologic studies [4].

Aptamers are single-stranded nucleic acids with defined tertiary structures for selective binding to target molecules by utilizing complementary shape interactions. The secondary structures of aptamers can be predicted from their sequences [5]. Many programs have been developed to identify and study aptamer binding motifs, including AptaTRACE [6] for identifying sequence-structure binding motifs in high-throughput systematic evolution of ligands by exponential enrichment (HT-SELEX) data. Aptamotif [7] and APTANI [8] also identified sequence-structure motifs in SELEX-derived aptamers using an ensemble-based approach. As only a small fraction of the sequence is involved in direct contact with the target, aptamer truncation can be used to find the minimum sequence while maintaining selective binding activity. Ultimately, aptamer truncation will help to reduce the material cost of aptamer synthesis, enable material quality assurance and prevent unexpected toxicity [9,10,11,12]. Many studies have demonstrated effective aptamer truncations in different ways. Truncations on the stem-loop regions showed a significant increase in the binding affinity for the VEGF-165 [13]. Aptamer acquisition from a random region has shown successful identification of 15 nt [14] or 20 nt [15] aptamers. Moreover, a 14 nucleotides aptamer was reported as the smallest functional unit of the transferrin receptor aptamer with enhanced binding affinity to the transferrin receptor [16].

Vimentin is an extracellular matrix protein that is part of the intermediate filament protein family [17]. Overexpression of vimentin may be associated with increased metastatic capacity through the epithelial to mesenchymal transformation (EMT) of ovarian tumor cells [18]. Studies have shown that increased expression of vimentin correlates to decreased survival rate in a variety of cancers such as colorectal, cervical, breast, gastric, and non-small cell lung cancers, to name a few [19,20,21]. There are limited studies, however, exploring the relationship between vimentin expression and ovarian tumor prognosis. A recent study by Szubert et al. [22] showed a prolonged overall survival rate with an increased rate of vimentin expression in the ovarian tumor cells. While this relationship is contrary to that seen in other tumor pathologies, it remains evident that a relationship exists between vimentin and overall tumor prognosis, and a marker for vimentin would serve as an invaluable tool in assessing tumor prognosis, as well as potentially serving as a marker for targeted molecular imaging. Currently, there are aptamers against vimentin for isolation of circulating tumor cells undergoing epithelial mesenchymal transition [23]. Zamay et al. used aptamer NAS-24 which binds to vimentin for intracellular targeting of mouse ascites adenocarcinoma cells in vitro and in vivo [24]. We have identified two phosphorothioated aptamers, thioaptamers, V3 and V5 that have high affinity and specificity binding to vimentin through our innovative morphology-based tissue aptamer selection (Morph-X-Select) method [25]. The secondary structure prediction suggests that those aptamers would form 3–4 stem-loop structures. The originally selected V3 and V5 aptamers were 74-mer in length and had high affinities to vimentin (Kd = 42.46 nM, Kd = 95.22 nM, respectively). As the recognition-based binding activity between the DNA aptamer and vimentin protein is dependent on the secondary structure of the vimentin aptamer, we further analyzed predictive secondary structures of the aptamers to identify sequence features and patterns by Mfold [5], and conducted a few truncations on the stem-loop regions to achieve improved binding affinity with the truncated aptamers.

## 2. Results

### 2.1. Identification of Potential Aptamer Binding Motifs

**Figure 1:**
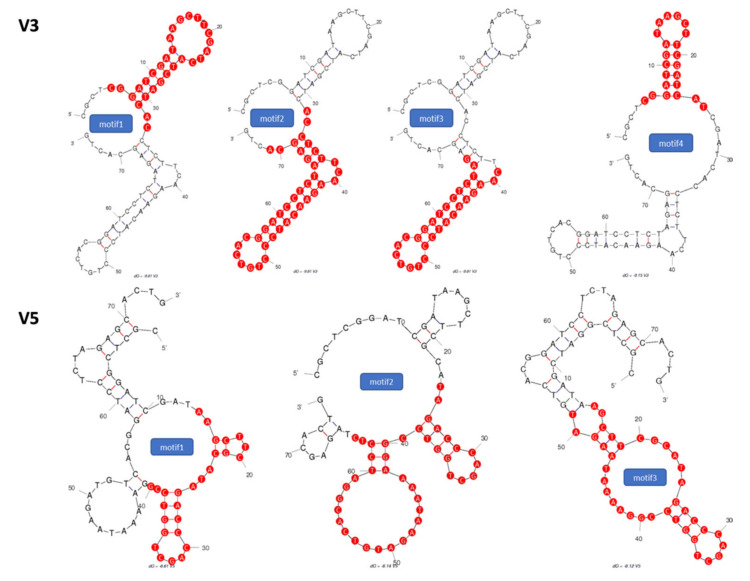
Secondary structures of selected (**V3**) and (**V5**) thio-aptamers. Proposed binding motifs are shown in red.

To improve upon the binding affinity of the vimentin binding aptamers that were identified in previous work by Wang et al. [25], the secondary structures of two vimentin binding aptamers (V3 and V5) were analyzed by Mfold to estimate short binding motifs. As the stems and loops structures are central to target molecule recognition, we conducted aptamer truncation on the stem-loop regions and generated seven truncated motifs. The selected secondary structures of V3 and V5 with their respective tentative binding motifs are shown in Figure 1. After identifying the binding motif sequences, we synthesized those binding motifs individually with modification of monothioated phosphates on their 3′ sides of adenines.

### 2.2. Screening of Synthesized Aptamer Motifs

**Figure 2:**
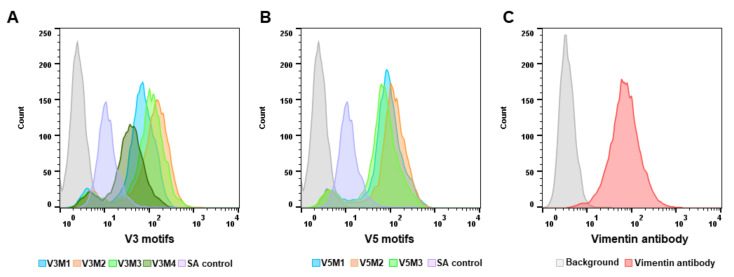
Screening of synthesized aptamer motifs. Synthesized biotinylated motifs of V3 and V5 were incubated with IGROV cells. Fluorescein isothiocyanate (FITC) conjugated streptavidin is used to detect biotinylated motifs. The binding affinity of the aptamer motifs was assessed based on the fluorescence intensity using flow cytometry. Histogram graphs demonstrated the fluorescence intensity of V3 aptamer motifs (**A**), and V5 aptamer motifs (**B**) binding to IGROV cells. Vimentin expression was evaluated and confirmed using an anti-human vimentin antibody before screening the binding affinity of motifs (**C**). A scrambled aptamer was used as a control.

After synthesis and purification of all seven aptamer motifs (Table S1), the binding affinity of each motif was assessed using the ovarian tumor IGROV cell line. Vimentin expression of IGROV cells was evaluated and confirmed using anti-human vimentin antibody before screening the motifs binding affinity (Figure 2C).

Afterward, cells were incubated with the vimentin-aptamer motifs and their binding affinity was assessed using flow cytometry. Based on the fluorescence intensity of each motif binding to the cells, the V3 aptamer motif 2 (V3M2) (Figure 2A) and the V5 aptamer motif 2 (V5M2) (Figure 2B) that have the highest fluorescent intensity of each group were selected for future experimentations and their sequences are listed in Table 1.

### 2.3. Binding Affinity of Selected V3M2 and V5M2

**Figure 3:**
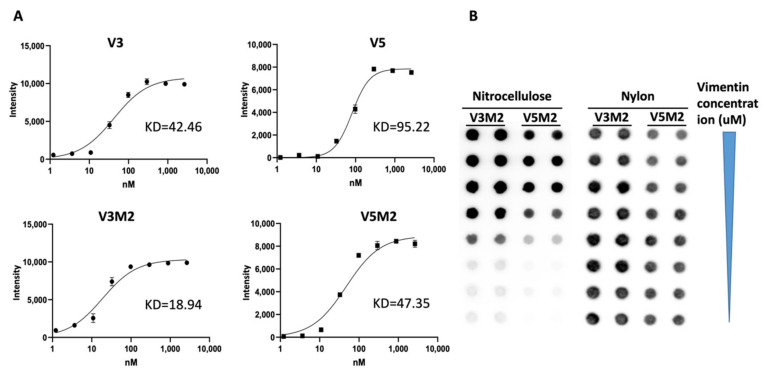
The binding affinity of V3, V5, selected motifs and their equilibrium dissociation constant. Filter-binding assays were performed with the biotinylated V3, V5, V3M2, V5M2 thioaptamers and purified vimentin protein. Chemiluminescent detection of spot intensities on the nitrocellulose membranes was used to quantitate the thio-aptamer binding affinity. (**A**) Saturation binding curves were generated and the equilibrium dissociation constants, Kd, were calculated from the equation Y = Bmax × X/(Kd + X), assuming a single binding site. Bmax represents the maximum binding capacity of aptamer bound to vimentin protein. X is the protein concentrations and Y is the calculated spot intensity. (**B**) Representative spot image of biotinylated V3M2 and V5M2 binding with vimentin protein retained on the nitrocellulose membrane. Non-binding motifs stained on a nylon membrane.

To quantitatively evaluate the binding affinity of the selected V3M2 and V5M2, filter-binding assays were performed with vimentin protein. The spot intensities of chemiluminescent signals were measured to establish saturation binding curves as shown in Figure 3A, lower panel. The equilibrium dissociation constants, Kd, were derived from these curves and are determined as V3M2 = 18.94 nM and V5M2 = 47.35 nM, respectively. Representative spot image demonstrated that biotinylated V3M2 and V5M2 bind with vimentin protein retained on the nitrocellulose membrane, while non-binding aptamers stained on nylon membrane (Figure 3B). Furthermore, the binding affinity of the short motifs V3M2 and V5M2 to vimentin protein was better than the binding affinity of the original V3 and V5 thioaptamers (Kd = 42.46 nM, Kd = 95.22 nM, respectively) (Figure 3A, upper panel).

### 2.4. Dose Response of V3M2 and V5M2

**Figure 4:**
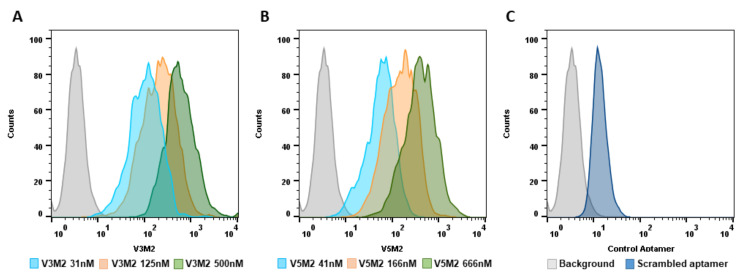
Dose-dependent binding of selected motifs. Biotin conjugated V3M2 and V5M2 were incubated with vimentin-expressing IGROV cells at various concentrations and followed by streptavidin-FITC staining. Their binding affinity was analyzed by flow cytometry. Histograms presenting the fluorescence intensity above the background were shown for V3M2 (**A**) and V5M2 (**B**). A scrambled control aptamer with non-specific and low binding affinity is also assessed (**C**).

To quantitatively evaluate the binding affinity of selected V3M2 and V5M2 further, vimentin expressing IGROV cells were incubated with biotin conjugates motifs at various concentrations (31, 125, 500 nM for V3M2, and 41, 166, 666 nM for V5M2), followed by streptavidin-FITC staining. The binding affinity of V3M2 and V5M2 at various concentrations was determined by mean fluorescence intensity using flow cytometry. Histograms of the fluorescence intensity above the background for V3M2 and V5M2 are shown in Figure 4A,B, respectively. These data demonstrated that the binding of both V3M2 and V5M2 to IGROV cells occurs in a dose-dependent manner. Moreover, at the same aptamer concentration, V3M2 showed better binding with higher fluorescence intensity than the binding of V5M2 to IGROV cells. This result is also consistent with the results of filter binding analysis in Figure 3.

### 2.5. Validation of Selected Motifs with Human Cells

**Figure 5:**
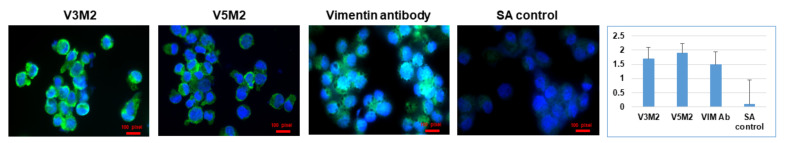
Validating specific binding of selected motifs with the human cell line. Vimentin-expressing IGROV cells were incubated with biotinylated V3M2, V5M2, anti-human vimentin antibody (VIM Ab) or scrambled control (SA) aptamer. Fluorescein isothiocyanate (FITC) conjugated streptavidin was used to detect biotinylated aptamers. The binding proficiencies were determined by fluorescence intensity using fluorescence microscopy. Fluorescence intensity is quantified by normalizing the fluorescence intensity of pixel per area (intensity of motif/intensity of DAPI) and presented as a bar graph with mean ± SE of three replicates. Hoechst 33,342 was used to stain the cell nuclei (blue).

To evaluate the binding efficiency and specificity, the performance of V3M2 and V5M2 binding to IGROV cells were compared with anti-human vimentin antibodies. Vimentin expressing IGROV cells were incubated with either V3M2, V5M2, or anti-human vimentin antibodies. The binding proficiency of V3M2, V5M2, or vimentin antibody was examined by fluorescence microscopy and quantified by normalizing fluorescence intensity of pixel per area (intensity of motif/intensity of DAPI) and are presented as a bar graph with mean ± SE of three replicates (Figure 5).

Both V3M2 and V5M2 demonstrated similar binding affinity to vimentin expressing IGROV cells, compared to the binding intensity of vimentin antibody. Specific bindings of V3M2 and V5M2 were confirmed by minimal fluorescence detection from scrambled control aptamer incubated with IGROV cells.

### 2.6. Validation of Selected Motifs with Human Ovarian Tumor

**Figure 6:**
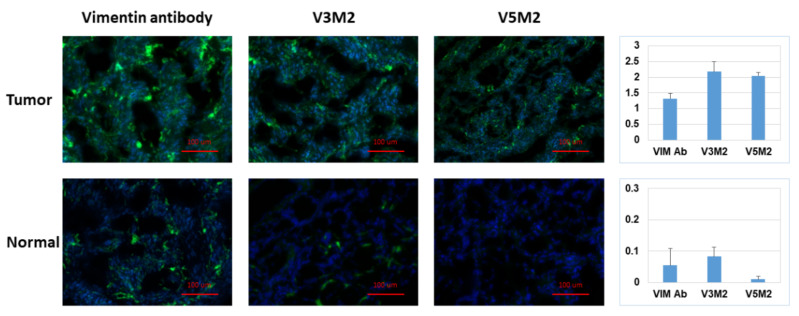
Detection of vimentin expression in human ovarian tumor tissue. Tissue sections of human ovarian tumor or normal ovarian tissue were incubated with biotinylated V3M2 or V5M2 at a concentration of 250 nM, followed by streptavidin-FITC to detect their binding affinity. Anti-human vimentin antibody was also used as a positive control for both ovarian tumor tissue and normal ovarian tissue. Images are representative of three samples of ovarian tumor or normal ovarian tissue. Fluorescence intensity is quantified by normalizing the fluorescence intensity of pixel per area (intensity of motif/intensity of DAPI) and presented as a bar graph with mean ± SE of three replicates. Hoechst 33,342 was used to stain the cell nuclei (blue).

After confirming aptamer binding in IGROV cells, the binding of the aptamers was further assessed in human ovarian tissue samples. Each aptamer was evaluated in both normal ovarian tissue samples and ovarian tumor tissue samples as displayed in Figure 6. The top row represented the strong binding of V3M2, V5M2 and anti-vimentin antibodies in human ovarian tumor tissue, while the bottom panel represented the weak binding of V3M2, V5M2 and anti-vimentin antibody in normal human ovarian tissue. All aptamers in this assay were used at a concentration of 250 nM. Images are representative of three samples of ovarian tumor or normal ovarian tissue. Fluorescence intensity is quantified by normalizing the fluorescence intensity of pixel per area (intensity of motif/intensity of DAPI) and presented as a bar graph with mean ± SE of three replicates. This observation is consistent with other studies that reported increased vimentin expression in various tumor cell lines and tissues including ovarian cancers [22], endometrial cancer [26] and many other tumors [27].

## 3. Discussion

Aptamer truncation is an important approach to lower the material cost of synthesis, improve on-target binding, and reduce non-target interactions of an aptamer. Based on secondary structure prediction, one can identify potential aptamer binding motifs as a way of truncating the sequence while maintaining selective binding activity. As the stems and loops structures are central to target molecule recognition, truncation on the stem-loop regions has demonstrated improved accessibility of targets to the aptamer, resulting in stronger aptamer-target binding [13]. Therefore, we conducted aptamer truncation on the stem-loop regions based on analysis of their predicted secondary structures of our identified V3 and V5 aptamers, and generated seven truncated motifs. The shortened motifs with fewer thiophosphate modifications can also reduce the non-specific binding against both target and non-target proteins compared with the normal phosphodiester bond [28]. By screening the binding affinity of the seven truncated motifs with vimentin expressing IGROV cells, we selected V3M2 and V5M2 based on their highest fluorescence intensity among truncations of V3 and V5 groups, respectively. When examining the sequences and secondary structures of the V2M2 and V5M2, we found both of them are the longest sequence in their truncated groups. Considering the stem-loop structures of V3M2 and V5M2, it is possible that the longer sequence contributes to the bigger loop and leads to better binding to the target protein. In fact, Armstrong et al. reported that the highest binding affinities were observed with full exposure of the aptamer sequence in the loop, while duplex formation reduced binding affinity most likely due to the thermodynamics of DNA base pairing [29]. This implies that the optimal motif selection might be to choose those motifs that have a long sequence size of the hairpin loop to accommodate both stem interactions and loop stability.

Although vimentin is mainly found in fibroblast, endothelial cells, macrophages, and lymphocytes, collective evidence suggest that the aberrant expression of vimentin in epithelial cancer cells is related to the epithelial–mesenchymal transition (EMT) and associated with tumor initiation, invasion, metastasis, and resistance to therapy [30,31]. Therefore, we used the ovarian tumor samples to assess the binding of V3M2 and V5M2 to vimentin expression ovarian tumors. We detected a strong signal of our selected motifs binding to ovarian tumor tissue compared with the normal ovarian tissue. The binding affinity of V3M2 and V5M2 to the extracellular matrix protein vimentin is comparable to that of anti-human antibodies in both cell and tissue binding evaluations. Truncations on the stem-loop regions demonstrated a significantly increased binding affinity (about 50% reduction of Kd values for both V4M2 and V5M2, Figure 3) to vimentin protein compared to that of the full-length aptamer sequences V3 and V5. Moreover, Tanaka et al. demonstrated intravenous administration of mono-thioated aptamer against E-selectin in mice did not cause overt hematologic, organs, and immunologic responses [32]. Therefore, we do not expect any significant toxicity of our selected mono-thioated aptamer motifs V3M2 and V5M2 for future applications.

The ability to modify and customize these aptamers with ease during their synthesis expands the potential applications when compared to antibodies with similar targets. By identifying and synthesizing potential binding motifs and validating their binding proficiency to the target of interest, the customizability and the overall performance of the aptamers have been enhanced. One potential future application for these aptamer motifs is to conjugate the aptamers to a synthetic dye and utilize the aptamer-dye conjugate as a tracer. This would allow for more specific tumor imaging to aid in monitoring tumor progression, as PET/CT is becoming increasingly useful in determining therapeutic efficacy in the field of oncology. It has recently been reported that up to forty percent of cancer patients have modified their treatment following analysis of tumor progression via PET/CT [4]. A study by Wu et al., demonstrated conjugation of a radionuclide tracer to an aptamer targeting a biomarker for glioblastoma multiforme [33]. They were able to show uptake of the aptamer-^118^Re conjugate in the tumor via a single-photon emission computerized tomography (SPECT) imaging after injection of the sample into the tail vein of a mouse model. If the vimentin binding aptamer motifs could be similarly conjugated to a radionuclide dye, the specificity of tumor imaging with elevated vimentin expression tumors could be greatly improved.

Other potential applications include conjugation of the vimentin-binding aptamer motifs to nanoparticles for both diagnostic evaluations and therapeutic interventions. A study with an aptamer designed to bind to prostate-specific membrane antigen (PSMA) conjugated the aptamer to superparamagnetic iron oxide (SPIO) nanoparticles [34,35]. The PSMA aptamer-nanoparticle conjugates were able to both enhance the magnetic resonance imaging signal and serve as delivery vehicles for drug therapy to treat prostate cancer. Another study used an epidermal growth factor receptor (EGFR) specific aptamer conjugated to hollow gold nanospheres (HAuNS) to target an EGFR positive tumor cell line [36]. Using SPECT/CT the authors were able to demonstrate greater and faster tumor cell uptake of the aptamer-HAuNS conjugate compared to antibody-HAuNS conjugates, suggesting a promising aptamer-based image guided drug delivery mechanism for EGFR positive head and neck cancers. Having a validated vimentin-binding aptamer with improved binding affinity and increasing relevance for vimentin as a biomarker for solid tumors, the potential applications for the vimentin binding aptamer motifs are plentiful.

## 4. Materials and Methods

### 4.1. Reagents

The nuclear stain used was Hoechst 33,342 (AnaSpec Inc., Fremont, CA, USA). The vimentin antibody used for cellular and tissue binding assays was a goat anti-human vimentin antigen affinity-purified polyclonal antibody (R&D Systems, Minneapolis, MN, USA). The secondary antibody was donkey anti-goat IgG, fluorescein conjugated (R&D Systems) and normal Goat IgG Control, Purified Goat IgG, (R&D Systems). The dye used for both cellular and tissue binding assays was streptavidin-FITC (BD Biosciences, San Jose, CA, USA). The blocking reagent used for tissue binding was Universal Blocker in TBS from Thermo Scientific (Waltham, MA, USA).

### 4.2. Aptamer Sequence and Structure Prediction

Based on the nucleotide sequences we identified against vimentin [25], the secondary structures of the ssDNA thio-aptamers were predicted using the Mfold web server (http://www.unafold.org/mfold/applications/dna-folding-form.php, accessed on September 2021). Using the DNA folding form, all possible secondary structures were approximated based on Watson-Crick base pairing and the most thermodynamically stable structures were selected. The initial sequences were selected as linear at a temperature of 22 °C and ionic concentration of 0.1 mM of Na^+^, 0.1 mM of Mg^2+^, computing only fold configurations within 5% of the minimum free energy, and considering a maximum number of 50 folds with no limit to the maximum distance between paired bases.

### 4.3. Aptamer Synthesis

According to the secondary structures of V3 and V5 predicted by Mfold, seven potential binding motifs were identified, and their sequences were synthesized using standard phosphoramidite chemistry on Expedite 8909 oligo synthesizers (Thermo Fisher Scientific, Waltham, MA, USA), as previously described [25]. The aptamer motifs were conjugated with 5′-biotin during synthesis to aid in purification and characterization. After synthesis, the aptamer motifs were purified using HPLC under reversed phase conditions on a Hamilton PRP-1 column using 100 mM triethylamine acetate (TEAA, pH 8.6) as the loading buffer and an acetonitrile gradient to elute the oligonucleotides.

### 4.4. Filter Binding Assay

The equilibrium binding constants of our selected motifs were examined by filter binding assay. The biotinylated V3M2 and V5M2 were incubated with varying concentrations of human vimentin protein in 10 μL of 20 mM Tris buffer (150 mM NaCl, pH 8.0) for 2 h at room temperature. After incubation samples were diluted to 100 μL with Tris buffer, transferred to the dot-blot apparatus and filtered under vacuum onto nitrocellulose membranes, which retain the vimentin with any bound V3M2 or V5M2. The amount of biotinylated V3M2 or V5M2 retained at each spot was determined by chemiluminescent detection using the Chemiluminescent Nucleic Acid Detection Module (Thermo Scientific) following the manufacturer’s instructions. The chemiluminescent signals were collected on a ChemiImager (ProteinSimple, San Jose, CA, USA). Image analysis and quantification of spot intensities were performed using ImageJ (version 1.53c) [37]. Binding analysis was based on the spot intensities on the nitrocellulose membranes with subtraction of background intensity from all the data points. Saturation binding curves were generated using GraphPad Prism (version 9.2.0) with curve fits assuming a single binding site. Using nonlinear regression, the equilibrium dissociation constants, Kd, were calculated from the equation Y = B_max_ × X/(Kd + X). B_max_ represents the maximum binding capacity of aptamer bound to vimentin protein. X is the vimentin protein concentration and Y is the calculated spot intensity.

### 4.5. Cell Binding Assay

Ovarian cancer IGROV cells (ATCC, Manassas, VA, USA) were maintained in RPMI-1640 medium supplemented with 10% fetal bovine serum (FBS) and 0.1% gentamicin sulfate. All cell culture reagents were purchased from Life Technologies (Grand Island, NY, USA). Cells were maintained at 37 °C in a 5% CO_2_ incubator with 95% humidity. All experiments were performed at 70–80% cell confluence. IGROV cells were seeded at a density of 2 × 10^5^ cells per well of an 8-well Nunc Lab-Tek II chamber slide with removable wells (Thermo Fischer Scientific, Waltham MA, USA). After fixing with 4% formaldehyde, cells were incubated with blocking buffer for 30 min to block unspecific binding of the aptamers.

Prior to cell binding assay, the aptamers were heated at 95 °C for five minutes and cooled slowly at room temperature to ensure the formation of the favored secondary structure. The respective aptamer was added to each well at the desired concentration and incubated for one hour at 37 °C. The cells were washed and streptavidin-FITC was added with a 1:500 dilution for a total volume of 250 µL per well for one hour. After nuclei counterstaining with Hoechst 33,342 (Thermo Fisher), the extent of aptamer binding to the cells was assessed by fluorescence microscopic analysis (TE2000-E, Nikon, Melville, New York, NY, USA). The relative binding affinity of aptamers was determined by the fluoresce intensity detected from the cells. An anti-human vimentin antibody was used as a positive control.

### 4.6. Aptamer Flow Cytometry

After trypsinization, IGROV cells were fixed with 4% paraformaldehyde and blocked with a universal blocking buffer (Thermo Fisher Scientific). Vimentin expression in IGROV cells was evaluated and confirmed using an anti-human vimentin antibody. V3M2 and V5M2, or scrambled control aptamer were incubated with IGROV cells at 22 °C for 1 h. After washing to remove excess aptamers, fluorescein-labeled streptavidin was incubated with cells for 30 min at room temperature. The binding of V3M2 or V5M2 to the targeted cells was measured by the fluorescence intensity with FACScalibur flow cytometry (BD Biosciences).

### 4.7. Tissue Binding Assay

Human ovarian tissue samples were collected at the University of Texas M.D. Anderson Cancer Center (MDACC) at the time of surgical intervention. Tissue samples were embedded in optimal cutting temperature compound (Thermo Fischer Scientific, Waltham, MA, USA). Tissue sections with 5–10 um thickness were mounted on a positively charged Super Frost glass slide (Thermo Fischer Scientific, Waltham, MA, USA). The slide was fixed and dehydrated with cold acetone for immunofluorescence staining. Sections of frozen human ovarian tumor or normal ovarian tissue were first incubated with Universal Blocker blocking buffer in TBS (Thermo Fisher Scientific, Waltham, MA, USA) and then incubated with 250 nM V3M2, V5M2 or scrambled aptamer for 2 h at room temperature with subsequent washing. The streptavidin-FITC labeled secondary antibody was incubated with tumor or normal ovarian tissue for 60 min in the dark at room temperature. Anti-human vimentin antibody was used as a positive control for specific detection of vimentin expression. Hoechst 33,342 (Thermo Fisher Scientific, Waltham, MA, USA) was used to counterstain nuclei. The binding of V3M2 and V5M2 to vimentin expression of ovarian tissue was assessed by fluorescence microscopic analysis (Nikon TE2000-E, Melville, New York, NY, USA).

## 5. Conclusions

In summary, we have identified two specific truncated aptamer motifs (V3M2 and V5M2) with successful binding to the extracellular matrix protein vimentin of IGROV cells and human ovarian tumor tissue. Truncations on the stem-loop regions demonstrated a significantly increased binding affinity to vimentin protein compared to the binding proficiency of the full-length aptamer sequences V3 and V5.

## Figures and Tables

**Table 1:** Selected short binding motifs.

Name	Sequences
V3M2 (40 mer)	5′-ACCTCTTCAAGAACATCCCTGTCACGGATCCTCTAGAGCA-3′
V5M2 (41 mer)	5′-TAGACCCAGCTGGTCCGGAAAATAAGATGTCACGGATCCTC-3′
Scrambled Control (40 mer)	5′-CCCACTTATCGTCCCTTAATGAGTTTACTCGCACACCGGA-3′

Adenines that have monothioated phosphates are shown in red.

## Data Availability

The data generated or analyzed during the study are included in the article.

## Supplementary Materials

The following are available online. Table S1: Short binding motifs of V3 and V5.
