# Supplementary material for: Selection and Characterization of Vimentin-Binding Aptamer Motifs for Ovarian Cancer
Source: Molecules. 2021 Oct 28;26(21):6525. doi: 10.3390/molecules26216525 (PMC8588432; doi:10.3390/molecules26216525)
Supplement: Supplementary file 1 [file molecules-26-06525-s001.zip › molecules-1394096-supplementary.pdf]

Supplementary Materials:

Table S1. Short binding motifs of V3 and V5.

Name	Sequences
V3M1 (29mer)	5′-CGGATCGATAAGCTTCGATCATCGATCAC-3′
V3M2 (40mer)	5′-ACCTCTTCAAGAACATCCCTGTCACGGATCCTCTAGAGCA-3′
V3M3 (29mer)	5'-CAAGAACATCCCTGTCACGGATCCTCTAG-3′
V3M4 (22mer)	5'-CGGATCGATAAGCTTCGATCAT-3′
V5M1(27mer)	5′-AAGCTTCGCATAGACCCAGCTGGTCCG-3'
V5M2 (41mer)	5′-TAGACCCAGCTGGTCCGGAAAATAAGATGTCACGGATCCTC-3'
V5M3 (37mer)	5'-AGCTTCGCATAGACCCAGCTGGTCCGGAAAATAAGAT-3'

Adenines that have monothioated phosphates are shown in red.
